# Supplementary figures and images for: Single-cell profiling of kinase substrate phosphorylation by single-molecule imaging
Source: PLoS One. 2026 Jun 5;21(6):e0350695. doi: 10.1371/journal.pone.0350695 (PMC13240859; doi:10.1371/journal.pone.0350695)

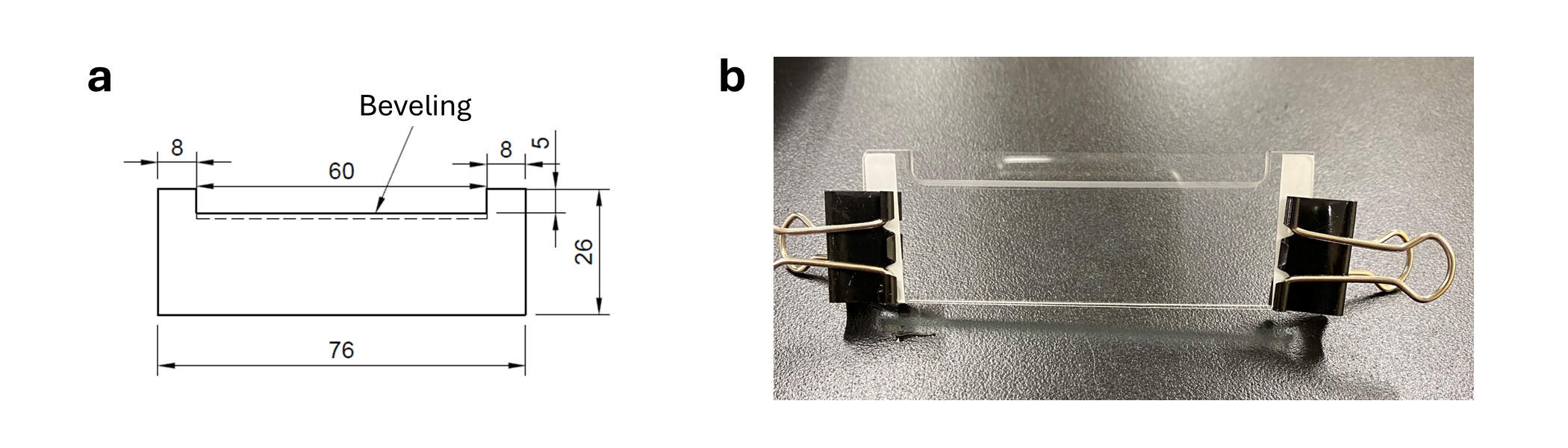

Supplement: S1 Fig — (a) Design of the notched glass plate for glass slide gel preparation. (b) Assembly of the silane-coated glass slide and the notched glass plate. (TIF) [file pone.0350695.s001.tif]

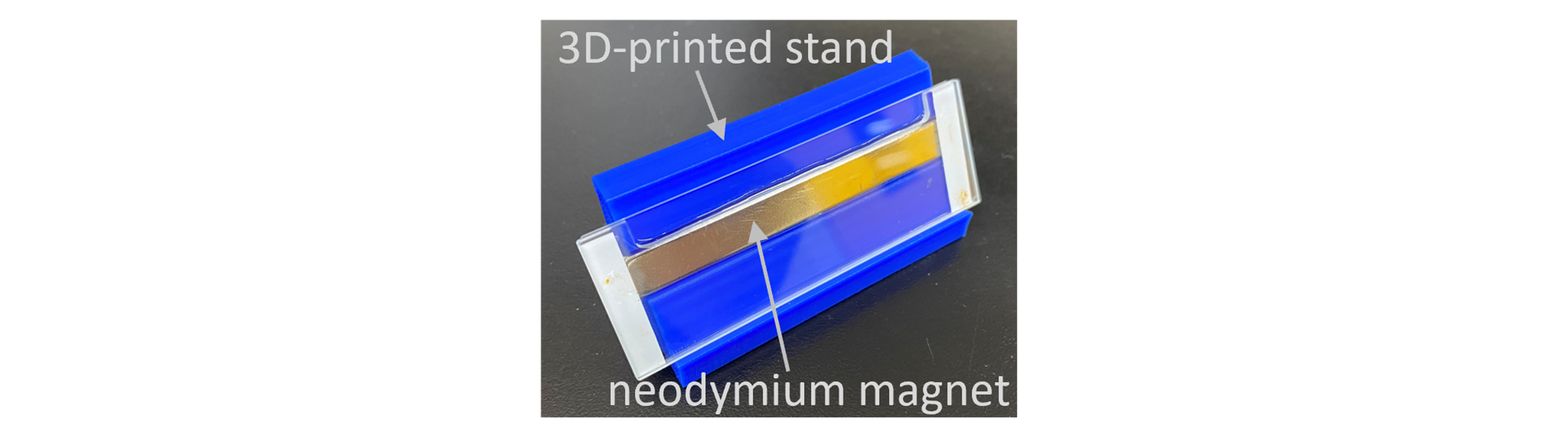

Supplement: S2 Fig — (TIF) [file pone.0350695.s002.tif]

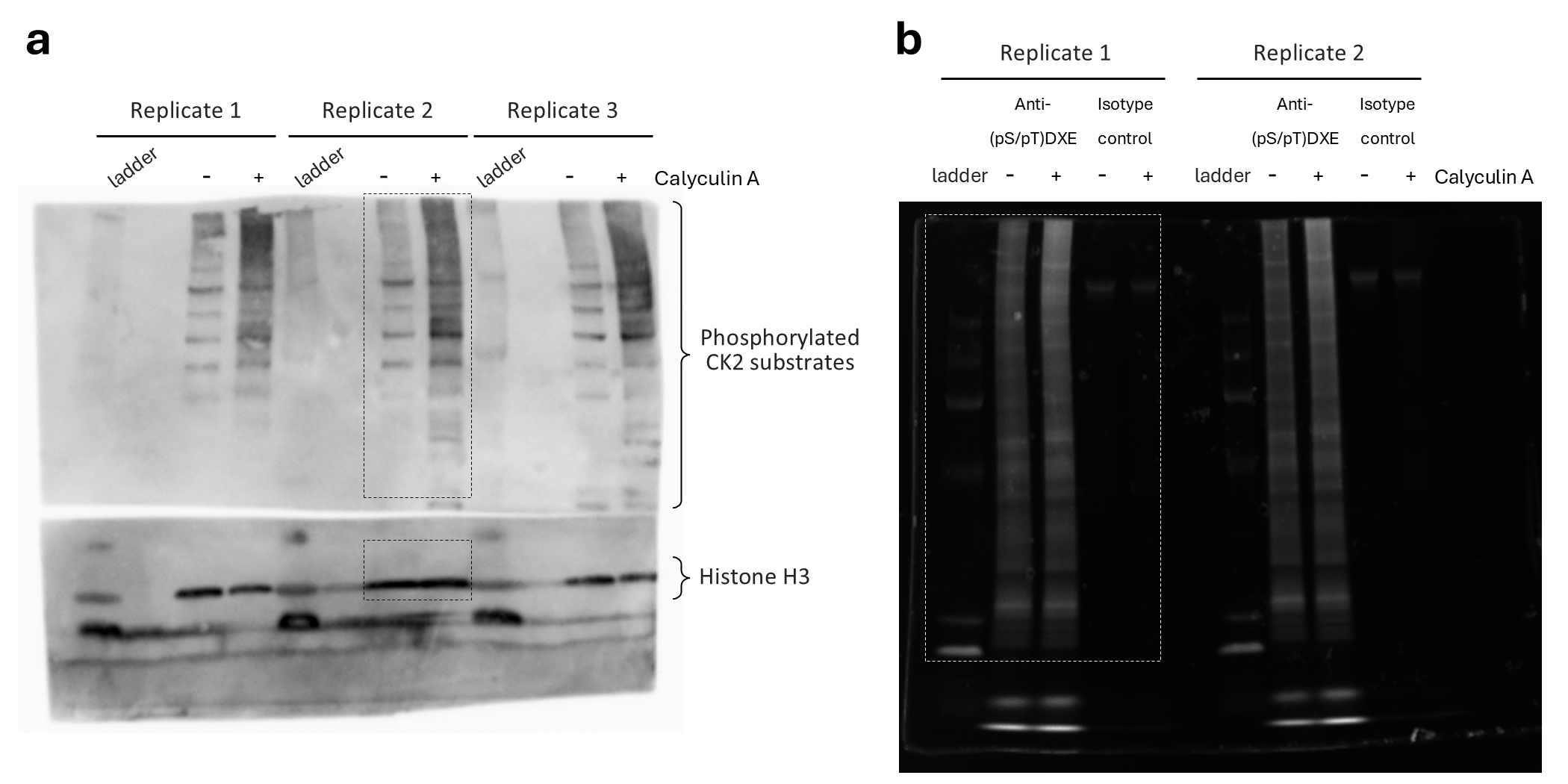

Supplement: S3 Fig — Uncropped images of (a) the western blot shown in Fig 2a and (b) the gel shown in Fig 2b. The regions presented in the main figures are indicated by dashed boxes. (TIF) [file pone.0350695.s003.tif]

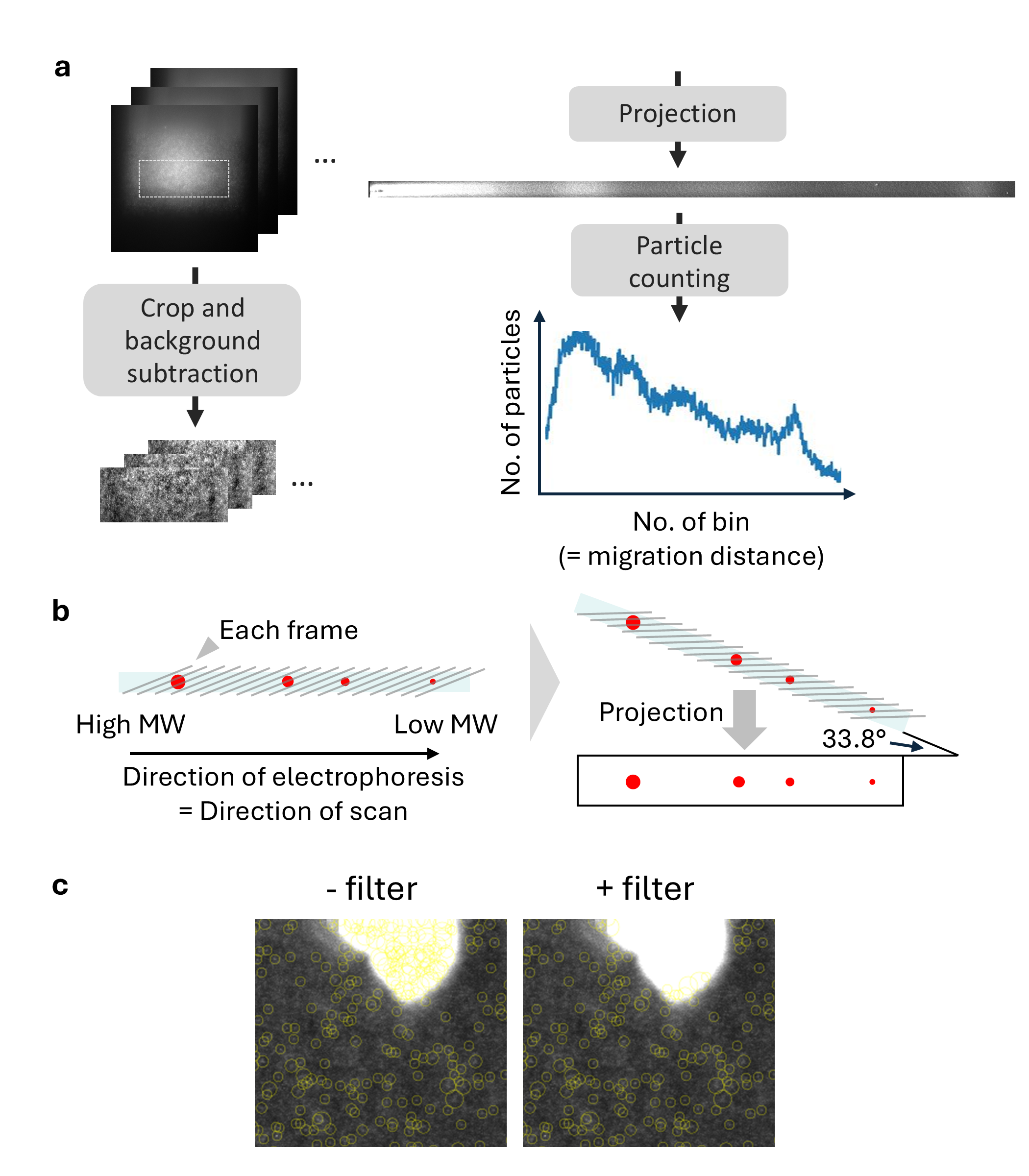

Supplement: S4 Fig — (a) Workflow of the image analysis pipeline used to convert a series of fluorescence images obtained from glass slide gel scanning into protein molecule profiling data. (b) Schematic illustration of a glass slide gel scan (left) and the projection of the acquired images (right). (c) Representative particle detection results with and without signal intensity and size filtering. Detected particles are indicated by yellow circles. (TIF) [file pone.0350695.s004.tif]
