# Supplementary material for: Single-cell profiling of kinase substrate phosphorylation by single-molecule imaging
Source: PLoS One. 2026 Jun 5;21(6):e0350695. doi: 10.1371/journal.pone.0350695 (PMC13240859; doi:10.1371/journal.pone.0350695)
Supplement: Attachment — Submitted filename: Response to Reviewers.pdf [file pone.0350695.s009.pdf]

Hidaka et al., response to the reviewers' comments:

We sincerely appreciate the reviewers for their thorough and constructive assessments of our manuscript, which have significantly contributed to improving our study. We have addressed each comment and have revised the manuscript accordingly. Below, we provide a detailed point-by-point response to each concern. The reviewer's remarks are presented in ***bold italics***, followed by our responses. Modifications in the revised manuscript are highlighted in red font.

Our response to Reviewer #1:

In this manuscript, authors address the challenge of analyzing low abundant phosphoproteins at single-cell level. Building upon their previous studies on the PISA microscope and single-cell PAGE-PISA (referenced on bioRxiv [10, 11]), they present a methodological approach to profile kinase substrate phosphorylation in individual cells. The workflow involves fluorescence labeling of the proteome, enrichment of phosphorylated substrates containing specific amino acid motifs via immunoprecipitation using motif-recognizing antibodies, and detection of single molecules through gel electrophoresis coupled with a custom-built imaging system. Using HeLa cells, the authors analyze CK2 substrates and demonstrate that their method enables single-cell-level comparison of phosphorylation patterns, with phosphoproteins separated by molecular weight prior to single-molecule detection.

We sincerely thank the reviewer for this clear and accurate summary of our work.

1. Reference papers for foundational methodological work: Given that the foundational methodological works [10, 11] are available as preprints, clarification regarding their current publication status would help contextualize the robustness and maturity of the approach described here.

We thank the reviewer for this helpful suggestion. Reference [10] is currently under preparation and has not yet been published. Reference [11] has recently undergone a second revision and has been resubmitted. In the revised manuscript, we have updated the reference information to reflect the current status of reference [11] in the Reference section.

2. Single-cell variability and experimental error control: While the included specificity controls (e.g., isotype control immunoprecipitation and BSA immunoprecipitation) support the selectivity of the assay, the manuscript would benefit from a clearer explanation of how experimental variability at the single-cell level was controlled. If internal references (e.g., spike-in controls or other normalization strategies) were used to monitor technical fluctuations between samples, please clarify. Otherwise, a brief discussion of how technical variability was distinguished from biological heterogeneity would further strengthen the robustness of the conclusions.

We thank the reviewer for this important comment. In this study, we used homogenized samples diluted to the single-cell level (pseudo-single-cell samples) as a control to assess technical variability between measurements. Because these samples are derived from a homogeneous lysate, they are expected to exhibit identical phosphoprotein profiles; thus, any observed variation primarily reflects technical fluctuations in the experimental workflow.

By comparing these pseudo-single-cell samples with true single-cell measurements, we observed that the variability in true single-cell samples was greater than that attributable to technical noise alone. This supports the conclusion that the additional variability arises from biological heterogeneity between individual cells. We have now clarified this rationale in the Results section of the revised manuscript (lines 207–216).

3. Figure 1C - labeling: For clarity, please specify in the figure legend what the labels "1" and "2" represent in Figure 1C (e.g., initial bright-field imaging (1) followed by fluorescence image scanning (2)).

We thank the reviewer for this helpful suggestion. In the revised manuscript, we have changed the labels in Figure 1C to "Step1: Bright field imaging" and "Step2: Fluorescence image scanning".

4. Figure 1D - scale bar: It would improve clarity to include an additional scale bar in the zoomed panel of Figure 1D.

We thank the reviewer for this suggestion. In the revised manuscript, we have added an additional scale bar to the zoomed panel in Figure 1D.

5. Statistical analysis (Figure 2E): While the visual comparison in Figure 2E indicates greater variability in single-cell samples, providing a statistical evaluation of variability would strengthen the interpretation.

We thank the reviewer for this important suggestion. To provide a more statistical evaluation of variability, we performed an additional analysis by calculating the standard deviation at each bin for both pseudo-single-cell and single-cell samples and visualizing the distributions as histograms.

This analysis shows that single-cell samples exhibit greater variability compared to pseudo-single-cell samples, consistent with the initial observation from the phosphoprotein profiles. The corresponding histograms have been added as Figure 2f, and the discussion about the analysis of variability between pseudo-single-cell and single-cell samples has been modified in the Result part of the revised manuscript (lines 214–216).

6. Figure organization (Figure S1): Figure S1 provides methodological clarification that appears conceptually earlier in the experimental workflow than Figures S2 and S3. However, it is currently cited later in the text. Adjusting the placement of Figure S1 (e.g., integrating into Figure 1B or revising the citation order) may improve the logical flow of the manuscript.

We thank the reviewer for this helpful observation. We identified an error in the figure numbering in the original manuscript. Although Figures S2 and S3 were cited in lines 127–128, the correct references should have been Figures S1 and S2. This has been corrected in the revised manuscript.

7. Reproducibility and accessibility of gel electrophoresis setup (Figure S2): Additional details regarding the fabrication and availability of the gel prepation and electrophoresis setup (e.g., the 3D-

printed gel casts) would improve reproducibility. Are the design files publicly available, or can they be requested from the authors?

We thank the reviewer for this suggestion. In the revised manuscript, we have provided additional details on the fabrication and accessibility of the electrophoresis setup. Specifically, we included a design schematic of the notched glass plate for gel preparation in Figure S1. In addition, the 3D model data for the magnetic stand used for bead pellet loading and for the electrophoresis chamber have been made available as Supplementary File 2 and Supplementary File 3, respectively. We have also expanded the Materials and Methods section to include further details on the fabrication of the electrophoresis chamber (lines 416-420).

8. Microscopy step size and potential signal loss: The rationale for selecting the microscopy step size should be clarified. Specifically, how was it determined relative to the expected protein size or diffusion characteristics? Providing clarification on whether signal loss between optical sections, particularly for smaller proteins, is expected to be negligible would further strengthen confidence in the detection strategy.

We thank the reviewer for this insightful comment. In our fluorescence image scanning, samples were scanned along the direction of electrophoresis with a step size of 10.73 µm. The thickness of the light sheet in our PISA microscopy setup was experimentally measured to be FWHM = 3.8 µm, and in practice, the laser intensity is sufficient to detect fluorescence from regions thicker than 6 µm (reference [10]).

Because the PISA microscope illuminates and images optical slices at a tilted angle of 33.8°, the effective spacing between adjacent optical sections is reduced to approximately 10.73 µm × sin(33.8°) ≈ 6 µm. This spacing is comparable to the detectable thickness of the illuminated region, indicating that signal loss between adjacent optical sections is negligible. Therefore, the chosen step size is sufficient to capture nearly the entire sample volume.

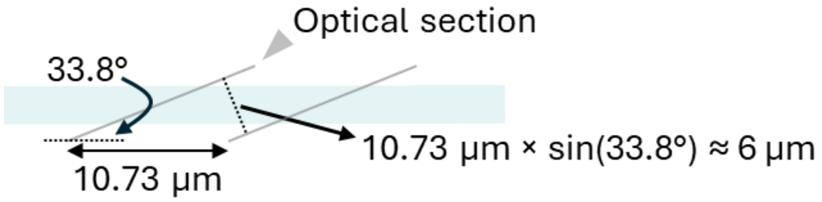

This rationale has now been clarified in the Materials and Methods section of the revised manuscript. (line 441-444)

Overall, the study presents a technically sound and innovative approach. The suggested revisions primarily concern clarification and statistical strengthening rather than fundamental methodological concerns.

We thank the reviewer again for the constructive feedback and the positive assessment of our work. We believe that the revisions made to the manuscript, together with the responses provided above, have addressed all concerns and improved the clarity and rigor of the study. We hope that the manuscript is now suitable for publication in *PLOS ONE*.

Our response to Reviewer #2:

This manuscript describes a novel and creative method for single-cell phosphoprotein profiling integrating fluorescence labeling, motif-specific immunoprecipitation, miniaturized SDS-PAGE, and single-molecule imaging using PISA microscopy. The approach is technically innovative and enables visualization of cell-to-cell heterogeneity in CK2 substrate phosphorylation. The system appears highly sensitive and could represent a useful platform for studying kinase signaling at single-cell resolution. The authors' discussion of potential future coupling with mass spectrometry to enable protein identification further strengthens the conceptual scope of the work.

However, in its current form, the method does not permit identification of the proteins contributing to each detected peak, which substantially limits biological interpretation. The standalone utility of the method therefore requires clearer articulation. In addition, several methodological, reproducibility, and interpretational issues should be addressed prior to publication in PLOS ONE.

We sincerely thank the reviewer for the constructive evaluation of our manuscript, and we also appreciate the reviewer's important comments regarding the current limitation in protein identification and the need to more clearly articulate the standalone utility of the method. In response, we have revised the manuscript to better clarify the scope and applications of the approach, and to more explicitly discuss its current limitations.

1. Protein Identification and Scope of Interpretation

• Although the authors discuss potential coupling with mass spectrometry, the current study does not allow identification of the proteins underlying each molecular weight region. This limitation should be more explicitly stated and discussed in the manuscript.

We apologize for not making this point clear that the current system cannot identify proteins underlying each molecular weight region. We added a new paragraph in the Discussion section (lines 298-303) to state and discuss this limitation separately from the potential coupling with mass spectrometry.

• The method relies on immunoprecipitation using an antibody against the (pS/pT)DXE motif. This approach captures only a subset of CK2 substrates and may also pull down substrates of other kinases sharing this motif. The manuscript should clearly acknowledge:

o That only motif-containing substrates are assessed.

o That the detected proteins cannot be definitively assigned as CK2 substrates without orthogonal validation.

o That substrates lacking this motif are not captured by the current system.

Greater clarity here would prevent overinterpretation of the data.

We thank the reviewer for this important comment and apologize for the overrepresentation of the (pS/pT)DXE motif as a strictly CK2-specific motif in the original manuscript. We revised the introduction of CK2 kinase (lines 156-158) to describe CK2 substrate specificity more accurately, noting that CK2 preferentially phosphorylates motifs containing multiple acidic residues, with a consensus sequence of pS/pT-D/E-X-D/E, where the +3 position is most critical, followed by the +1 position.

We added a new paragraph in the Discussion section to acknowledge the limitation more broadly that only motif-containing substrates are assessed and substrates of other kinases sharing the same or similar motif might contaminate in the analysis, making orthogonal validation required to definitively assign the detected proteins as substrates of target kinases (lines 287-290).

2. BSA-like Peak in Isotype Control (Figure 2C)

A peak corresponding to the migration position of BSA appears in the isotype control condition in Figure 2C. The authors should clarify:

• The origin of this peak.

• Whether it reflects residual nonspecific binding, antibody contamination, or background labeling.

• Why this peak appears in both treated and untreated samples.

This is important for assessing background levels and specificity.

We thank the reviewer for this important comment and apologize for the lack of clarity in the original manuscript regarding the peaks observed in the isotype control in Figure 2C.

The prominent peak at a migration distance of approximately 3500 μm is attributed to full-length antibody, while smaller peaks around 6000 μm likely arise from fragmented antibody species. These signals are observed in all samples because the antibodies were not crosslinked to protein G beads and can therefore co-elute with captured proteins during sample preparation.

We note that the strong signal from full-length antibody interferes with the quantification of sample-derived signals in that region. Therefore, for subsequent analyses, we restricted quantification to regions beyond the full-length antibody peak.

We apologize for not clearly describing this in the original manuscript. This explanation has now been added to the revised manuscript (lines 190–197).

3. Pseudo-Single-Cell vs. Single-Cell Heterogeneity (Figures 2E and 2F)

In Figure 2E, one treated single-cell replicate appears to be a potential outlier. Because the heterogeneity analysis and PCA in Figure 2F heavily rely on 2E dataset, an outlier could disproportionately influence conclusions.

The authors should:

• Confirm whether this sample reflects biological variability or technical artifact.

• Consider repeating the experiment or increasing replicate number.

• Re-evaluate PCA and heterogeneity analysis after addressing this issue.

Given that the primary conclusion of the manuscript is the detection of cell-to-cell heterogeneity, robustness of this analysis is essential.

We thank the reviewer for this critical comment. We agree that one of the calyculin A-treated single-cell samples shows significantly lower signal compared to others, and it is possible that this reflects a technical artifact, such as unsuccessful single-cell sorting.

To address this concern, we analyzed a different set of calyculin A-treated samples and compared the variability by generating histograms of standard deviation at each bin for both pseudo-single-cell and single-cell samples (Fig. 2E and 2F in the revised manuscript). This analysis consistently showed greater variability in single-cell samples compared to pseudo-single-cell samples, particularly after calyculin A treatment, supporting our original conclusions.

We also repeated the PCA analysis using the updated dataset and observed similar trends (Fig. 2G): untreated and treated samples are separated along the primary principal component, reflecting overall phosphorylation levels, while treated samples further segregate into subpopulations along PC3. Peaks at approximately 4,000, 6,500, and 11,500 µm show strong positive loadings on PC3.

In accordance with this revision and Reviewer 1's comment #5, we have updated the description of the single-cell variability and PCA analyses in the manuscript (lines 207–216 and 224–229; Fig. 2E–G).

4. Concordance Between Profiles in Figures 2C, 2D, and 2E

When comparing profiles across Figures 2C, 2D, and 2E, the migration patterns and signal distributions do not appear fully concordant.

The authors should comment on:

- ***The reproducibility of the profiling method across experiments.***
- ***Sources of variability between runs (e.g., gel preparation, labeling efficiency, IP efficiency).***
- ***Quantitative metrics of reproducibility, if available.***

Clarifying inter-experimental variability would strengthen confidence in the method.

We thank the reviewer for this important comment regarding reproducibility and inter-experimental variability. We acknowledge that the current workflow involves multiple experimental steps, among which protein separation using a very thin (~100 µm) miniaturized polyacrylamide gel is particularly critical. The quality of gel preparation strongly affects electrophoretic mobility, separation efficiency, and overall reproducibility between experiments. We anticipate that reproducibility and resolution can be further improved by implementing more stringent control of gel preparation conditions, including temperature, humidity, and deoxygenation, as well as by optimizing gel composition. We have added this discussion to the revised manuscript. (lines 272-278)

Although we have not established formal quantitative metrics of reproducibility across experiments, we performed an additional analysis to assess variability in pseudo-single-cell samples. Specifically, using the dataset shown in Figure 2C, we generated histograms of the standard deviation at each bin, analogous to the analysis presented in Figure 2F (the figure below). This analysis showed that the variability in these pseudo-single-cell samples is comparable to or lower than that observed in Figure 2F, suggesting that the variability observed in true single-cell samples exceeds inter-experimental variability.

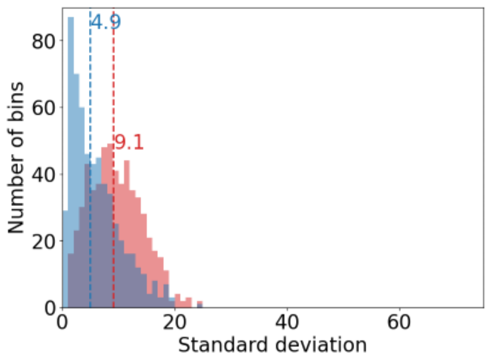

Fig. Histograms of the standard deviation for each bin, calculated from the phosphorylation profiles shown in Fig. 2c. Red indicates calyculin A–treated samples, and blue indicates untreated samples. Dashed lines represent the median values.

5. Fluorophore-to-Protein Stoichiometry and Quantitative Interpretation

The method relies on NHS-ester labeling, which depends on lysine number and accessibility. This raises important quantitative considerations:

• How do the authors account for variability in labeling stoichiometry across different proteins?

• Is labeling assumed to be proportional to protein abundance?

• How might differential lysine content affect comparison across molecular weight regions?

We thank the reviewer for raising these important points. As noted, fluorescence labeling with NHS-ester dyes depends on the number and accessibility of lysine residues, and proteins with fewer accessible lysines may be labeled less efficiently. Consequently, labeling efficiency is not uniform across proteins and in general, larger or more basic proteins tend to be labeled more efficiently than smaller or more acidic proteins. Therefore, comparisons are most reliable within the same molecular weight region across different samples, rather than between distinct regions. We have clarified this limitation in the Discussion section of the revised manuscript (lines 280–285).

Additionally, when multiple proteins of similar molecular weight co-migrate, how is signal overlap addressed? These issues should be discussed explicitly, even if only as methodological limitations.

In addition, as discussed in the original manuscript, the current system has limited electrophoretic resolution, and multiple protein species may co-migrate within the same region, leading to overlapping signals. While resolution could be improved by extending the migration distance or optimizing gel composition, this remains a limitation of the present setup. We also note that variability in labeling efficiency due to differences in lysine accessibility may provide additional contrast between protein species within the same region.

However, this does not enable definitive identification and more accurate quantitative interpretation will require further development to integrate the current system with orthogonal approaches, such as mass spectrometry. We have expanded the Discussion section to more explicitly address these methodological limitations (lines 292–303).

Minor Concerns

1. Antibody Specificity

• The anti-(pS/pT)DXE antibody recognizes a motif shared among multiple proteins and is not exclusive to CK2 substrates. The manuscript should clarify that "CK2 substrates" are inferred based on motif recognition rather than direct kinase validation.

• The limitation that not all CK2 substrates contain this motif, and that other kinases may generate similar motifs, should be clearly stated.

We thank the reviewer for this important comment. We have revised the manuscript to avoid implying that the (pS/pT)DXE motif is exclusive to CK2 substrates and to clarify the limitations of motif-based inference. Specifically, we have made the following changes:

• In line 165, we revised "… (pS/pT)DXE motif present in a broad range of CK2 substrates …" to "… (pS/pT)DXE motif present in a subset of CK2 substrates …".

• We added the following sentence in line 165: "It should be noted that proteins detected with the anti-(pS/pT)DXE antibody are referred to as phosphorylated CK2 substrates in this study, although other kinases can occasionally phosphorylate the same motif."

• In line 180, we replaced "anti-CK2 substrates antibody" with "anti-(pS/pT)DXE antibody" for accuracy.

• We also added a new paragraph in the Discussion section explicitly acknowledging that only motif-containing substrates are assessed that substrates of other kinases with similar motifs may be captured, and that orthogonal validation is required to definitively assign kinase specificity (lines 287–290).

2. Materials and Methods – Immunoprecipitation of bulk samples

• Please list all antibodies used with Protein G beads (source and catalog number).

• The BSA control experiment shown in Figure 2C should be described in the Methods section.

We thank the reviewer for pointing out this omission and apologize for the lack of detail in the original manuscript. In the revised version, we have added information on all antibodies used for immunoprecipitation, including their sources and catalog numbers, in the Materials and Methods section (lines 350, 360-362). In addition, we have included a description of the BSA control experiment, specifying the amount and concentration of the BSA solution used (line 376-377).

3. Materials and Methods – Image Analysis

• The criteria used for thresholding and distinguishing true single-molecule foci from aggregates, bubbles, or dust should be described in greater detail.

• Inclusion of representative raw images (including excluded artifacts) in the supplementary material would improve transparency.

We thank the reviewer for this suggestion to improve transparency. We have added more details on the image analysis, particularly regarding the thresholding criteria for particle detection and the filtering of signals from impurities, in the Materials and Methods section (lines 464–471). In addition, we have included representative data showing particle detection before and after filtering in Fig. S4c. We have

also provided representative projection images and their overlays with detected particles from the samples analyzed in Fig. 2c as Supplementary File 1, along with the corresponding caption (lines 246–248).

Summary

This manuscript presents a technically innovative platform for single-cell phosphoprotein profiling. The method appears sensitive and capable of detecting heterogeneity at the motif level, and the discussion of future integration with mass spectrometry enhances its conceptual appeal.

However, clarification of key methodological limitations, quantitative assumptions, antibody specificity, reproducibility across experiments, and interpretation of the heterogeneity analysis is necessary. Addressing these issues would significantly strengthen the manuscript and make it suitable for publication in PLOS ONE.

We thank the reviewer again for the constructive and comprehensive evaluation of our work. We appreciate the recognition of the strengths of our approach, as well as the insightful comments regarding its limitations and interpretation. We believe these revisions have substantially improved the clarity and rigor of the study, and we hope that the manuscript is now suitable for publication in *PLOS ONE*.
