# Supplementary material for: Single-cell profiling of kinase substrate phosphorylation by single-molecule imaging
Source: PLoS One. 2026 Jun 5;21(6):e0350695. doi: 10.1371/journal.pone.0350695 (PMC13240859; doi:10.1371/journal.pone.0350695)
Supplement: Attachment — Submitted filename: Response_to_Reviewers_auresp_2.pdf [file pone.0350695.s010.pdf]

Hidaka et al., response to the reviewer's comments:

We sincerely appreciate the reviewers again for their careful assessments of our manuscript. We have addressed each comment and have revised the manuscript accordingly. Below, we provide a detailed point-by-point response to each concern. The reviewer's remarks are presented in ***bold italics***, followed by our responses. Modifications in the revised manuscript are highlighted in red font.

Our response to Reviewer #1:

I thank the authors for their detailed responses and the clarifications provided. The revised manuscript successfully addresses most of my previous concerns. However, there are a few remaining points that should be addressed before final publication:

1. Methodological Solidity: I note that reference [10] remains a preprint and has not yet been submitted for peer review, and reference [11] is also not yet formally published. As these works underpin key methodological aspects, their current status limit how solidly the present study can be assessed. I recommend the authors update these citations if they have since been accepted, or provide further justification for relying on unpublished work for core methods.

We thank the reviewer for this important comment. Reference [11], renumbered as reference [12] in the revised manuscript, has recently been accepted for publication, and we have updated the citation information accordingly.

Reference [10] has not yet been submitted for peer review. However, the key design principle underlying the PISA microscope—namely, the open-top configuration that enables volumetric imaging of laterally large samples by stage scanning—has been patented (Taniguchi and Nishimura: Patent JP6086366, US9880378, EP2983029). Moreover, through a licensing agreement with ZEISS, this design principle has been implemented in the ZEISS Lattice Lightsheet 7, the first commercially available open-top light-sheet microscope. To clarify the basis for the methodological approach proposed in this study, we have also added the relevant patent information as a new reference [11] in the revised manuscript.

2. Quantitative Validation of Variability: While Figure 2F visually indicates a shift in standard deviation, the manuscript still lacks a formal quantitative/statistical evaluation to confirm this difference. Briefly acknowledging in the Discussion that establishing robust quantitative metrics remains a challenge (as noted in the rebuttal) would provide a more transparent context for the results.

We thank the reviewer for raising this point related to the inter-experiment reproducibility and the reliability of observed cell-to-cell heterogeneity. As pointed out by the reviewer and mentioned in our previous rebuttal letter, we have not yet established robust reproducibility metrics across experiments and needs to increase replicate numbers for both the pseudo-single-cell and single-cell groups across independent experiments to establish such metrics. We clarify this limitation in the Discussion in the revised manuscript (lines 278–285).

3. Figure Quality: The figures provided require higher resolution to ensure clear representation and readability in the final layout.

Thank you for raising this concern regarding figure resolution. We confirm that all figures were submitted at high resolution (300 dpi). The reduced image quality observed in the review file appears to be due to compression during the review file generation process. The original high-resolution figures will be used for the final production layout.

4. Typological Errors: There are formatting issues in lines 69, 93, 96, and 444, where sentences appear to end prematurely before the referenced citations. Please review the manuscript for consistent punctuation and citation placement.

We thank the reviewer for pointing this out. We have thoroughly reviewed the manuscript and corrected the relevant formatting issues to ensure consistent punctuation and citation placement throughout the text. The corrections were made in lines 59, 69, 93, 96, and 451 of the revised manuscript.

Overall, the current version presents a potentially sound methodological approach, provided that the supporting references are formalized, and it correctly addresses the study's limitations. Once these minor technical and formatting issues are resolved, the manuscript will be in a much stronger position for publication.

We thank the reviewer again for the positive assessment and helpful comments. We believe these revisions have improved the manuscript and made it suitable for publication.
